# The Case of Joan of Arc: Was the Maid Insane?

**Published:** 1917-08

**Authors:** Arthur C. Jacobson

**Affiliations:** Brooklyn, N. Y.


### THE CASE OF JOAN OF ARC: WAS THE MAID INSANE?

**Arthur C. Jacobson, M.D.**
**Brooklyn, N. Y.**

"This is the voice which I seem to hear murmuring in my ears, like the sound of the flute in the ears of the mystic; that voice, I say, is humming in my ears, and prevents me from hearing any other. . . Then let me follow the intimations of the will of God." —Socrates: In Plato's Crito.

"There is a natural body, and there is a spiritual body," wrote Saint Paul to the Corinthians, enunciating a profound distinction which has guided all psychologists ever since. Only the psychologists treat Saint Paul's spiritual body, or soul, as merely a secondary personality.

Men like Andrew Lang think that what was divine in the Maid made itself intelligible to her ordinary consciousness, or workaday self, and led her to the fulfilment of a task which seemed impossible to men. This, of course, is the religious man's way of stating the matter. The psychologist would say that it was the Maid's secondary personality (or ordinary personalities) which guided the acts of her primary conventional self.

We can understand this matter of secondary personality better if we pause for a moment to consider the distinction between the conscious and the subconscious minds. Let us think of the subconscious mind in Graham's terms, as the cellar of human personality, at once a storehouse and a factory. It is a storehouse in the sense that within it are kept records of our experiences and memories. It is a factory in the sense that within it are manufactured all sorts of ideas, many of which issue in the shape of dreams or the so-called creations of genius.

Now this cellar of human personality, says Graham, has a trap-door, or perhaps several trap-doors. When they are open, during the hours either of sleep or waking, up rush the records and the products of the cellar, coloring our thoughts and acts according to their volume and character, and according to the nature of our environment.

That which transpires in the realm of the subconscious, or subliminal, represents the activities of the secondary self. We must think of a subliminal intelligence just as we think of a conscious, or supraliminal intelligence. Thus there is the mind of the natural body, and the mind, or soul, or spirit that modern scientists think of in connection with the secondary personality. When we speak of the primary self we mean the natural body and mind—by which an individual is ordinarily identified.

Now there are a good many cases on record of multiple personality. It seems that the Maid came under this category, for she appears to have been directed in all that she did by three advisers. She called them Saint Margaret, Saint Catherine, and the angel Michael. She said that one always remained with her, that one visited her often, and that the third was one with whom the others consulted.

When one takes into account the religious psychology of the times, there is nothing strange in Joan's assumption that the voices that spoke to her were those of saints and angels. The illusions in which she saw such saints and angels were visual phantasms prejected and dramatized by the subliminal self for supraliminal observation. If a modern psychologist heard such voices or saw such visions he would not call them the voices or apparitions of saint, but voice b, apparition c, illusion n, etc. An untutored peasant girl of the fifteenth century, thirteen years of age at the time of her first hallucinations (so-called) in other words at an age when the nervous spstem of a girl undergoes great biological disturbances, would naturally interpret the monitions emanating from her secondary personalities in terms different from the modern psychologist.

"Who was without visions in the Middle ages?" asks Michelet, in his "History of France." And taking into consideration the views of the age about good and evil spirits, one cannot produce any proof that the Maid was actually insane within our modern meaning of the term. Andrew Lang covers the point very well when he says that her experiences were not associated with insanity, the sagacity and veracity of the communications distinguishing them from the hallucinations of mad people. The phenomena were orderly, sane, wise and noble, or rather heroic. Joan typified unification of human powers. So far were her impulses from madness that they were wiser than sanity itself, as Myers strikingly puts the case.

Joan's supraliminal intelligence. which was of a high order, acted concurrently and co-ordinately with the promptings of her secondary personalities, if we take properly into account the issues and the beliefs of her day. Great ends were served. There was a happy mixture of subliminal with supraliminal faculties, and she utilized them in wider range than any of her contemporaries. In the case of the insane the sweet bells are out of tune and all is dissonance. The inter-relations of the primary and secondary selves are functionally inco-ordinate, and puerile or criminal ends are sought. The psychology of the Maid was supernormal, but not abnormal, and psychiatric criteria do not hold or even apply in her case. If they are made to apply to her, they must also be applied to Socrates, as Myers has pointed out, and we are reduced to absurdity.

It will readily be conceded that Socrates was a man of transcendent genius, yet he was guided in all things by his subliminal self, or Daemon, as it was then called. This "permanent type of sanity, shrewdness, physical robustness and moral balance," this Founder of Science himself, was, like Joan, condemned on the ground of his monitory voices. His Apology, says Myers, like Joan's, resolutely insisted on the truth of the very phenomena which were used to destroy him. Xenophon says: "As to his not worshipping the gods whom the city worshipers, what evidence was there of this? He sacrificed constantly, and obviously used the art of divination; for it was a matter of notoriety that Socrates said that the divine Providence gave him indications; and this indeed was the principal reason for accusing him of introducing new gods."

The voice decided him to action, or to abstention from action, which he professes always to have recognized as right and wise. The voice gave him knowledge unattainable by ordinary means (intuition?). The voice gave him clairvoyant powers, or extension of the ordinary range of sense.

In obedience to the voice, Socrates dissuaded his friends from expeditions which ultimately turned to their harm, in the case of the Athenian expedition against Syracuse. He also warned Timarchus of his impending assassination. The most noteworthy instance of the prompting of the voice was in his own case when accused of impiety. By it he was warned not to resist his persecution. The voice intervened to check him from preparing any speech in his own defense. After the passing of the sentence he did speak, however, and here follow his words:

"There has happened to me, O my Judges, a wonderful thing, for that accustomed divine intimation in time past came to me very many times, and met me on slight occasion, if I were about to act in some way not aright; but now this fate which ye behold has come upon me—this which a man might deem, and which is considered, the very worst of ills. Yet neither when I left my home this morning was I checked by that accustomed sign; nor when I came up hither to the judgment-hall, nor at any point in my speech as I spoke. And yet in other speeches of mine the sign has often stopped me in the midst. But now it has not hindered me in any deed or word of mine connected with this present business. What then do I suppose to be the reason thereof? I will tell you. I think it is that what has happened to me has been a good thing; and we must have been mistaken when we supposed that death was an evil. Herein is a strong proof to me of this; for that accustomed sign would assuredly have checked me, had I been about to do aught that was evil."

Socrates was subject to fits of abstraction, or ecstacy, sometimes lasting for hours. Once, for a day and a night, he was unresponsive to any outward stimulus, remaining fixed in deep contemplation. At such times his subliminal self was so near the surface that it temporarily predominated the whole man. Similar instances of wise automatism are familiar in the cases of other great geniuses, especially during creative moments, for example, Shelley and Hawthorne. In the genius there is a facile shifting of the gears of personality, distinguishing him from commonplace men. The absent-mindedness and tendency to reverie of the genius group serve in a rough way to identify them.

Joan first heard her voices at thirteen years of age. She was commanded to be a good girl, and to go often to church. After this the voice intervened frequently on all sorts of occasions.

At about the age of eighteen she received the final summons to fight for France.

At first the voice was accompanied by a light, afterward by figures of saints. These saints appeared to speak to Joan.

It seemed to her that she could both see and feel them. There is some reason to believe, however, that on one occasion she was deceived by a pageant gotten up by the courtiers for political ends. She asserted (evidence before the ecclesiastics, 1431) that several persons, including the Archbishop of Rheims and herself, had been an angel bringing a crown to the King. Michelet thinks that such a piece of trickery accounts for the Maid's singular evidence on this point. Lang thinks the Maid was speaking allegorically.

The voice, or voices, often came spontaneuosly; if they did not, she summoned them when needed by a simple prayer to God. They came as a rule during her waking hours, but sometimes roused her from sleep. The voice was not always fully intelligible, especially if she happened to be half awake or disturbed by a tumult. Sometimes her sublimal personality failed to externalize, clearly focussed, the figures of the saints for observation by her primary self.

The predictions of the voice were fulfilled; namely, that the siege of Orleans would be raised; that Charles VII. would be crowned at Rheims; that she herself would be wounded. Myers remarks as to these that about as much was fulfilled as an ardent and devoted mind might have anticipated—much indeed that might have seemed irrational to ordinary observers, but nothing which actually needed a definite prophetic power.

Myers makes the following significant comments on the case of Joan:

"Among all the messages given to Joan of Arc, there does not seem to have been one which fell short of the purest heroism. They were such commands as were best suited to draw forth from her who heard them the extreme of force, intelligence, virtue, of which she had the potency at her birth. What better can we desire as the guide of life? We need not assume that the voices which she heard were offspring of any mind but her own, any more than we need assume that the figures in which her brave and pious impulses sometimes took external form were veritable saints—the crowned Saint Margaret and the crowned Saint Catherine and Michael in the armoury of Heaven. Yet, on the other hand, we have no right to class Joan's monitions, any more than those of Socrates, as an incipient madness. To be sane, after all, is to be adjusted to our environment, to be capable of coping with the facts around us. Tried by his test, it is Socrates and Joan who should be our types of sanity; their difference from ourselves lying rather in the fact that they were better able to employ their own whole being, and received a clearer inspiration from the monitory soul within.''

Lang point out that until Joan told her tale at Rouen (1431) we hear not one word about saints or angels. She merely spoke of ''my voices,'' my ''counsel,'' ''my Master.'' She seems finally to have ascribed a distinctly religious character to her advisers because of the current influences. Such visions as Joan's possessed only one nomenclature in those days, and had to be discussed in current terms to be intelligible and acceptable.

Modern philosophers like Bergson have sought to persuade us to rely more implicitly upon our intuitions. This, after all, is only the highbrows' recognition that ''voices'' do speak to us. We fear, however, that unless the one spoken to be a Joan or a Socrates, it were best that our armies be headed by Haigs and our philosophies and ethics manufactured by academicians.

According to competent and credible contemporary testimony, Joan of Arc never experienced the periodical phenomenon peculiar to her sex. She always clung to male attire from the time of her visit to Robert of Bauricourt, at about the age of eighteen. She was tall and comely, with dark hair, and her voice is described as sweet. She was very reticent about speaking, and was readily moved to tears. Her demeanor was graceful and modest, and her countenance cheerful. She possessed very great physical endurance, and was skilful at riding and in the use of weapons. At handling artillery she was an expert. She wore the heaviest of armor. When wounded by an arrow above the breast at Orleans, she was afraid and wept. She tried to discourage gambling, and would not tolerate swearing. She had a special horror of plundering. When she saw the English soldiers lying wounded she had great compassion. When Glansdale, the English Captain, who had coarsely abused her, fell into the Loire trying to escape, and was drowned, she began to weep for his soul. Joan took a truly feminine dislike to the girls who followed the camp, and broke the sword of Fierbois over the back of a courtesan who was following her men-at-arms. She was skilful at sewing.

Her visions began at the age of puberty. It is possible that the suppression of the periodical phenomena to which we have alluded was in some way related to her psychic disturbances .

Masculinity and femininity, as we have seen, were curiously blended in her. Thus she was fitted to play her great part not only in point of physical adaption, but in point of sexual make up.

To the exacting demands of her extraordinary environment she was thus perfectly adapted. Taking into account the times, the issues and the prevailing belief in good and evil spirits, she must be regarded as sane, for if we hold sanity to consist mainly in adequate adaption to environment, who ever adapted himself better than Joan to a given set of trying circumstances and achieved greater ends?—The Medical Times, New York.

